# Is the inflammasome a potential therapeutic target in renal disease?

**DOI:** 10.1186/1471-2369-15-21

**Published:** 2014-01-23

**Authors:** Clare M Turner, Nishkantha Arulkumaran, Mervyn Singer, Robert J Unwin, Frederick WK Tam

**Affiliations:** 1 Imperial College Kidney and Transplant Institute, Hammersmith Hospital, Imperial College London, London, UK; 2 Bloomsbury Institute of Intensive Care Medicine, Division of Medicine, University College London, WC1E 6BT London, UK; 3 UCL Centre for Nephrology, Division of Medicine, Royal Free Campus and Hospital, University College London, WC1E 6BT London, UK

**Keywords:** Inflammasome, NLRP3, Renal disease, IL-1β, IL-18, PAMPs, DAMPs, P2X_7_R

## Abstract

The inflammasome is a large, multiprotein complex that drives proinflammatory cytokine production in response to infection and tissue injury. Pattern recognition receptors that are either membrane bound or cytoplasmic trigger inflammasome assembly. These receptors sense danger signals including damage-associated molecular patterns and pathogen-associated molecular patterns (DAMPS and PAMPS respectively). The best-characterized inflammasome is the NLRP3 inflammasome. On assembly of the NLRP3 inflammasome, post-translational processing and secretion of pro-inflammatory cytokines IL-1β and IL-18 occurs; in addition, cell death may be mediated via caspase-1. Intrinsic renal cells express components of the inflammasome pathway. This is most prominent in tubular epithelial cells and, to a lesser degree, in glomeruli. Several primary renal diseases and systemic diseases affecting the kidney are associated with NLRP3 inflammasome/IL-1β/IL-18 axis activation. Most of the disorders studied have been acute inflammatory diseases. The disease spectrum includes ureteric obstruction, ischaemia reperfusion injury, glomerulonephritis, sepsis, hypoxia, glycerol-induced renal failure, and crystal nephropathy. In addition to mediating renal disease, the IL-1/ IL-18 axis may also be responsible for development of CKD itself and its related complications, including vascular calcification and sepsis. Experimental models using genetic deletions and/or receptor antagonists/antiserum against the NLRP3 inflammasome pathway have shown decreased severity of disease. As such, the inflammasome is an attractive potential therapeutic target in a variety of renal diseases.

## Introduction

Inflammation is central to the pathogenesis of many renal diseases. The innate immune system, a first line defense against pathogens, is usually involved in the initiation and propagation of inflammation. It is activated by a series of germ-line encoded pattern recognition receptors (PRRs) that allow discrimination of ‘self’ from ‘non-self’ antigens. PRRs recognize conserved pathogen-associated molecular patterns (PAMPs) on invading organisms, or respond to host-derived danger-associated molecular patterns (DAMPs) released in response to stress, tissue injury, or cell death. Several classes of PRRs have been identified, including transmembrane Toll-like receptors (TLR), C-type lectin receptors (CLRs), the retinoic acid inducible gene-I (RIG-I) receptors, intracellular Nod-like receptors (NLRs), and the recently identified HIN-200 receptors [1-3]. Extracellular PAMPs and DAMPs are recognized by TLRs and CLRs, whereas NLRs and RIGs recognize intracellular molecular patterns (Table 1).

**Table 1:** Activators of the inflammasome

**Sterile activators**		**Pathogen activators (PAMPS)**			
**DAMPs**	**Environment derived**	**Bacteria derived**	**Virus-derived**	**Fungus-derived**	**Protozoa-derived**
ATP	Alum	Pore-forming toxins	RNA	β-glucans	Hemozoin
Cholesterol crystals	Asbestos	Lethal toxin	M2 protein	Hyphae	
MSU/CPPD crystals	Silica	Flagellin/rod proteins		Mannan	
Glucose	Alloy particles	MDP		Zymosan	
Amyloid β	UV radiation	RNA			
Hyaluronian	Skin irritants	DNA			

Activators of the inflammasome are divided into 2 categories: Sterile activators include host derived DAMPs and environment derived molecules, and pathogen activators include PAMPs derived from bacteria, virus, fungi and protozoa.

*Abbreviations*: *CPPD* Calcium pyrophosphate dehydrate, *DAMP* Damage-associated molecular pattern, *MDP* Muramyl dipeptide, *MSU* Monosodium urate, *PAMP* Pathogen associated molecular pattern.

PRRs are expressed primarily by innate immune cells, but also by endothelial and epithelial cells. The innate immune system is ‘primed’ by activation of PRRs by PAMPs or DAMPs, which leads to activation of numerous proinflammatory transcription factors, the best characterized being nuclear factor kappa-B (NF-κB) and activator protein-1 (AP-1), with subsequent transcription of multiple mediators (including cytokines and chemokines) and receptors.

A key mechanism responsible for the post-transcriptional processing and release of mature cytokines is formation of the inflammasome complex. The human genome encodes 23 NLR proteins broadly divided into NLRP (with a pyrin domain) and NLRC (with a caspase recruitment domain), a subset of which are capable of forming an inflammasome complex. This multiprotein cytosolic complex oligomerizes to provide a platform for processing and release of cytokines. Seven cytoplasmic receptors form an inflammasome complex: NLRP1 (NLR family, pyrin domain containing 1, also called NALP1), NLRP3 (also called NALP3 or cryopyrin), NLRP6, NLRP12, NLRC4 (NLR family, caspase recruitment domain (CARD) containing 4, also called IPAF), AIM2 (absent in melanoma-2), and RIG-1 (retinoic acid inducible gene 1). Of these receptors, the NLRP3 inflammasome is the best characterized.

## Review

### The NLRP3 inflammasome

This large multiprotein complex (>700 KDa) forms in response to diverse PAMPs, including lipopolysaccharide (LPS), peptidoglycan, bacterial DNA, viral RNA and fungi, and DAMPs such as monosodium urate crystals (MSU), calcium pyrophosphate dehydrate, cholesterol crystals, amyloid β, hyaluronan and, possibly, glucose [1] (Table 1).

**Figure 1:**
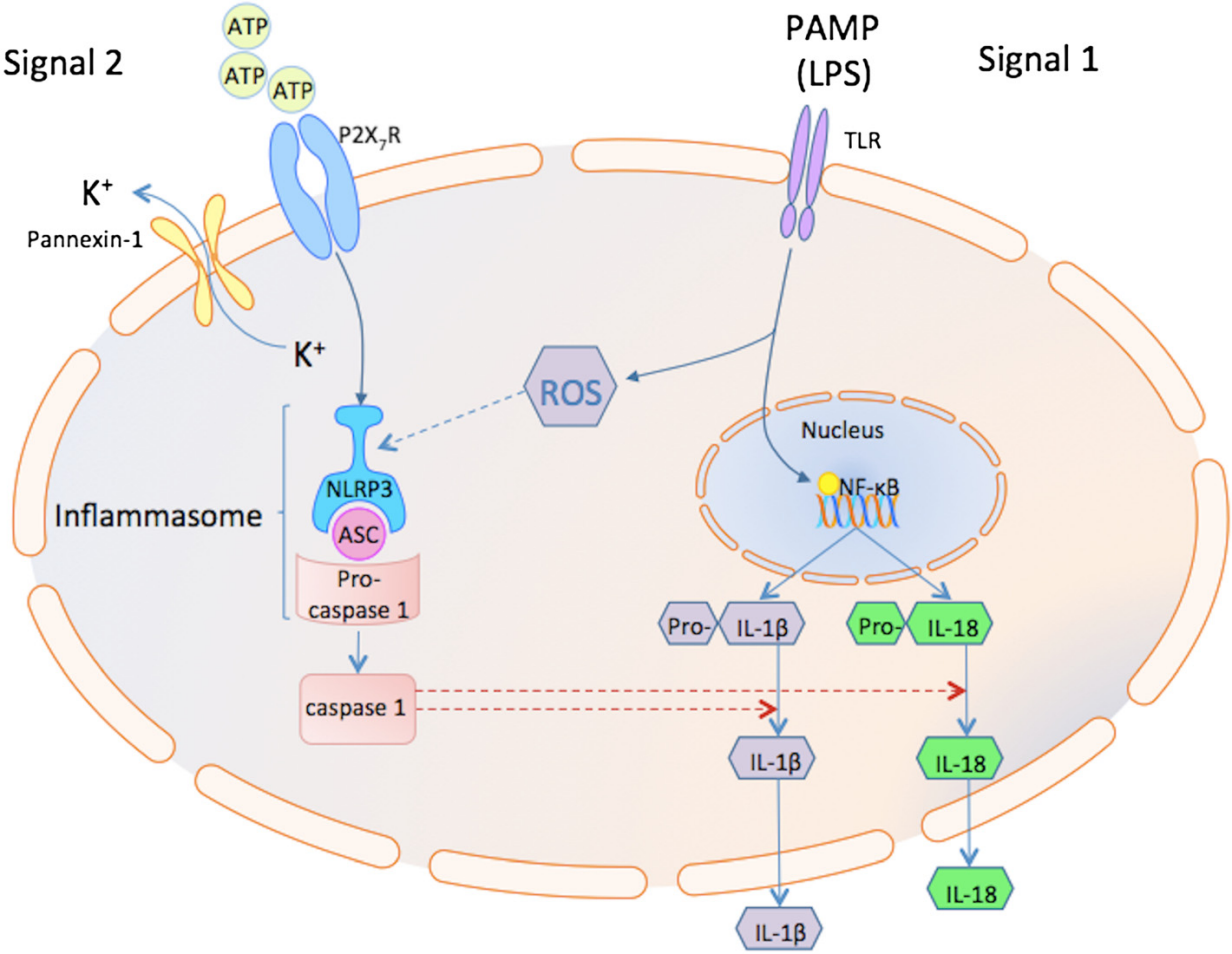
**Model of NLRP3 inflammasome activation.** NLRP3 is activated by a vast array of stimuli including extracellular pathogen PAMPs such as bacterial LPS via pattern recognition receptors (PRR) such as Toll-like receptors (TLR) and DAMPs. This comprises signal 1 and leads to synthesis of the cytokine precursor pro-IL-1β via NF-κB and other components of the inflammasome such as NLRP3 itself. Many of the known activators of the inflammasome generate ROS which can bind to NLRP3 and this appears necessary for its activation. Extracellular ATP binding to the P2X7 receptor (P2X7R) comprises signal 2. This promotes the recruitment and opening of the pannexin-1 pore channel which causes rapid K^+^ efflux, another event which appears necessary for NLRP3 activation. NLRP3 assembly occurs when, through its pyrin domain, NLRP3 binds to the pyrin domain on an ASC molecule which then binds to pro-caspase-1 via its CARD domain. This leads to cleavage of pro-caspase-1 and subsequent cleavage of pro-IL-1β and pro-IL-18 to their active forms. Abbreviations: DAMP, damage-associated molecular pattern; LPS, lipopolysaccharide; ROS, reactive oxygen species; PAMP, pathogen-associated molecular pattern; PRR, pattern recognition receptor; TLR, toll-like receptor; PYD, pyrin domain.

Priming of the cell (*signal 1*) by activation of PRRs results in NFκB -dependent transcription of pro-IL-1β and upregulation of NLRP3. Assembly of the NLRP3 inflammasome relies on the adaptor molecule ASC (Apoptosis-associated Speck-like protein containing a C-terminal caspase recruitment domain (CARD)). The ASC protein is composed of PYD (N-terminal pyrin domain) and CARD domains. The N-terminus of NLRP3 also contains a PYD that mediates homotypic binding with ASC via a PYD-PYD interaction. Through its CARD, ASC interacts with procaspase-1 leading to autocatalytic activation of caspase-1. This results in processing of pro-IL-1β and pro-IL-18 to their active forms (IL-1β and IL-18) and their release (Figure 1).

The cell surface P2X_7_ receptor (P2X_7_R) facilitates assembly of the NLRP3 inflammasome [4-6]. ATP released into the extracellular milieu during inflammation is a potent stimulus for P2X_7_R activation [7-9]. This results in formation of an ion pore and K^+^ efflux, with reduction in intracellular K^+^, a key step in inflammasome activation [10]. Activation of P2X_7_R by LPS and ATP results in MyD88-dependent NFκB activation (*signal 2*), and transcription of pro-IL-1β [11]. Following LPS priming of monocytes, P2X_7_R activation stimulates NADPH oxidase generation of superoxide anions, thereby facilitating NLRP3 activation [12].

### Other inflammasomes

**Figure 2:**
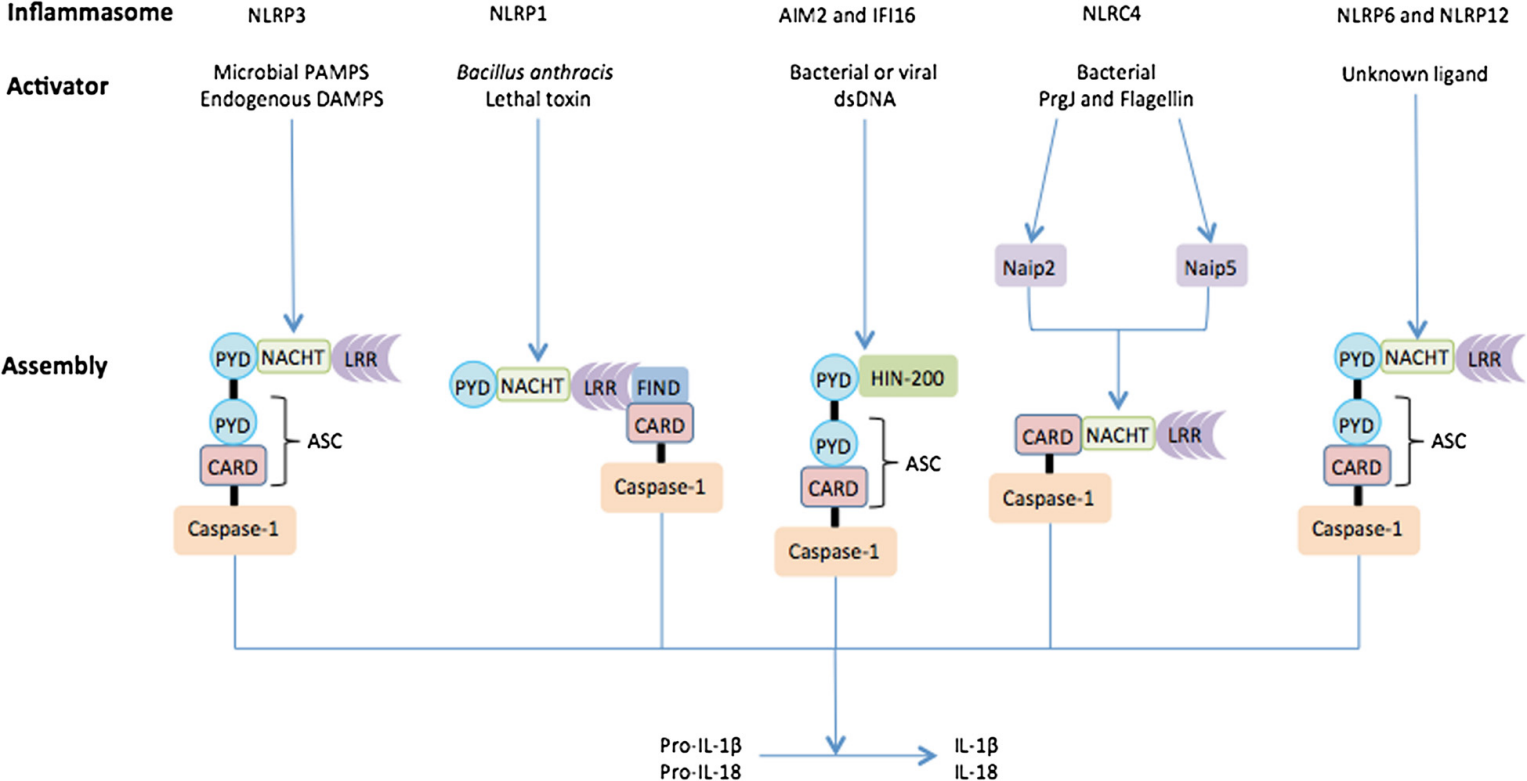
**Models for inflammasome activation and assembly.** The NLR family members and the HIN-200 proteins, AIM2 and IFI16, assemble inflammasome complexes. NLRs are characterised by a NACHT domain with or without an N-terminal PYD domain and a variable number of LRRs. AIM2 and IFI16 contain an N-terminal PYD domain followed by a DNA binding HIN-200 domain. The PYD domain of NLRP3, 6 and 12, AIM2 and IFI16 recruit the adaptor protein ASC via homotypic binding to its PYD domain allowing indirect recruitment of caspase-1 through interaction with the CARD domain. NLRP1 and NLRC4 directly recruit caspase-1 through a CARD domain. NLRC4 is activated by NAIP proteins bound to specific ligands, NAIP 2 binds to the bacterial rod protein PrgJ whereas NAIP 5 and 6 bind to bacterial flagellin. Activation of the inflammasome leads to maturation and secretion of IL-1β and IL-18 aswell as inflammatory cell death by pyroptosis. Abbreviations: AIM2, absent in melanoma 2; CARD, caspase recruitment domain; DAMP, danger-associated molecular pattern; FIND, domain with function to find; IFI16, Interferon-γ inducible protein 16; LRR, leucine rich repeat; NACHT, nucleotide-binding and oligomerization domain; NAIP, NLR family apoptosis inhibitor; NLR, Nod-like receptor, PAMP, pathogen associated molecular pattern; PYD, pyrin domain.

NLRP1 was the first inflammasome to be described and is activated following cleavage by the lethal toxin from *Bacillus anthracis*[13]. The NLRP1 inflammasome has its own CARD, so can bypass the requirement of the adapter molecule ASC for inflammasome activation (Figure 2). Cleavage by the anthrax toxin directly activates CARD, leading to activation of caspase-1 [13]. An alternative mechanism of NLRP1 activation is by the toxin inhibiting p38 mitogen-activated protein kinase and Akt kinase, leading to opening of the connexion channel for ATP release, resulting in P2X_7_R signaling [14]. There are similarities with the mechanism of activation of the NLRP3 inflammasome.

A second class of inflammasomes contains members of the PYHIN family, rather than NLRs. These are characterised by N-terminal PYD and C-terminal HIN-200 (hemopoetic interferon-inducible nuclear antigen with 200 repeats) DNA binding domains. Examples include AIM2 and Interferon-γ inducible protein 16 (IFI16) inflammasomes. These lack a CARD domain and require ASC for recruitment of pro-caspase-1 to form a stable inflammasome complex. The PYD domain interacts with the PYD domain of ASC. Following detection of bacterial or viral dsDNA, AIM2 and IFI16 inflammasomes assemble with subsequent secretion of IL-1β and IL-18 [15], which is severely impaired in mice deficient in AIM2 that are highly susceptible to *Mycobacterium tuberculosis* infection [16]. AIM2 can recognise self-DNA, but this is limited under steady-state conditions because of its cytosolic location. In conditions where self-DNA is not cleared from the extracellular compartment, it is likely that DNA can activate AIM2 and drive inflammation. Of note, HIN-200 proteins are considered a candidate locus for susceptibility to lupus [17]. In contrast to AIM2, IFI16 is located within the nucleus; the mechanism by which it discriminates between self and viral DNA in the nucleus is currently unknown.

The NLRC4 inflammasome interacts directly with pro-caspase-1 via homotypic CARD interactions, leading to processing of caspase-1. This inflammasome complex plays an essential role in the innate immune response to the bacterial proteins flagellin and PrgJ [18]. Direct binding of NLRC4 with flagellin or PrgJ has not been shown; however, the proteins of the NAIP family (NLR family, apoptosis inhibitor) act as immune sensors that can interact with, and control, NLRC4 activation. The NAIP2-NLRC4 complex associates with PrgJ, while the NAIP5-NLRC4 complex associates with flagellin [19]. This suggests that distinct NAIP proteins allow the NLRC4 inflammasome to differentiate among different bacterial ligands.

The NLRP6 inflammasome associates with ASC, inducing caspase-dependent processing and release of IL-1β. At the mRNA level NLRP6 is highly expressed in mouse liver, kidney and small intestine, and plays a central role in modulating inflammatory responses in the gut to allow recovery from intestinal epithelial damage, tumorigenesis, and in controlling the composition of the gut microflora to prevent colonization by harmful bacteria [20,21]. Data on NLRP6 and renal disease are limited and warrant further study.

The NLRP12 inflammasome is expressed in human myeloid cells. It acts as a negative regulator of inflammation by reducing NFκB activation and inhibiting chemokine expression through ATP hydrolysis [22]. NLRP12 also reduces NFκB activation by (i) TLR-signaling molecules MyD88, IRAK-1 (type I interleukin-1 receptor-associated protein kinase), and TRAF6 (TNF receptor (TNFR)-associated factor), and (ii) the TNFR signaling molecules TRAF2 and RIP1, but not the downstream NFκB subunit p65 [23]. NLRP12, like NLRP6, can contribute to the maintenance of intestinal epithelium, since mice deficient in NLRP12 are more susceptible to colonic inflammation and tumorigenesis [24].

### Processing of IL-1α, IL-1β, IL-18, and caspase-1

IL-1 is a key cytokine in many inflammatory diseases. Activation of MAPK and NFκB signal transduction pathways is central to the diverse actions of IL-1, which include production and/or release of nitric oxide (NO), cyclooxygenase-2 (COX-2) and superoxide products, and other pro-inflammatory mediators [25,26].

IL-1 has two biologically active isoforms, IL-1α and IL-1β, which bind to the same receptors [27,28]. Both are produced as 31 kDa precursors that are stored within the cytosol. Pro-IL-1α is constitutively expressed, whereas pro-IL-1β is transcribed in response to an inflammatory or infectious stimulus [25]. Various inflammatory stimuli engage with the PRR receptors of immune cells, activating MAPK and/or NFκB signalling cascades, and resulting in the synthesis of pro-IL-1β from its pro-IL-1β precursor, which is also stored within the cytosol.

IL-1α release is typically described as being passive, as a consequence of non-apoptotic cell death [29]. IL-1α processing depends on calpain protease activity [30]. The activation of calpain-like may be NLRP3-inflammasome/caspase-1 dependent or independent, depending on the type of NLRP3 agonist [31]. Caspase-1 knockout cells are unable to secrete IL-1α in response to soluble NLRP3 stimuli while caspase inhibitors have no effect, suggesting that the catalytic activity of caspase-1 is not required [32]. This protease- independent function of caspase-1 in the release of IL-1α is not well established.

Although IL-1α has similar biological activity in its precursor and cleavage product forms; in contrast, IL-1β is only active after cleavage to its 17 kDa mature form. Caspase-1 is crucial for processing of intracellular pro-IL-1β, although extracellular pro-IL-1β can be processed by several proteases, including serine proteinase 3 and the metalloproteinases MMP-2 and MMP-9 [33,34].

Caspase-1 is produced from the constitutively expressed 45 kDa cytoplasmic pro-enzyme, pro-caspase-1. This requires post-translational processing to form 20 and 10 kDa forms of active caspase-1 [34], and occurs following assembly of the NLRP3 inflammasome. Proteolytic activation of IL-1β occurs within the inflammasome complex. Mature IL-1β is released into the extracellular space by exocytosis or loss of membrane integrity [35].

Synthesis and release of IL-18 is closely linked to IL-1. IL-18 is a key mediator in the host response to infection and the inflammatory response [27,36]. It is also constitutively produced as a precursor, pro-IL 18 [36], which is cleaved by either caspase-1 or proteinase-3 into its active form released into the extracellular space along with mature IL-1β.

Contact with a DAMP or PAMP (‘signal 1’) alone is insufficient for extracellular release of IL-1β and IL-18. An additional stimulus (‘signal 2’), mediated by a variety of ligands including extracellular ATP, nigericin, bacterial toxins, hypotonic stress and T cells, is usually required for the extracellular release of active IL-1β and IL-18. However, the best-established stimulus for this post-translational processing and release is ATP, acting via the P2X_7_R [33,36].

### Effects of IL-1α, IL-1β, IL-18

Il-1β has diverse functions relating to its unique ability to regulate inflammation at both the nuclear and membrane receptor levels. Unlike other cytokines, the effects of IL-1β on lymphocytes are largely indirect, mediated by the induction of gene expression and synthesis of cyclooxygenase-1 (COX-2), prostaglandin-E2, platelet activating factor, NO, and IL-6 [25,26]. In turn, these mediators result in fever, vasodilation, hyperalgesia, and a repertoire of immune cell functions. IL-1α and IL-1β also act as co-stimulatory molecules of T cells with an antigen, and may contribute to T cell polarization (early Th17 differentiation *in vivo* and Th17-mediated autoimmunity) [37].

IL-1 induces angiogenesis via upregulation of VEGF. This mechanism is mediated primarily via the PI3-K/mTOR pathway in renal mesangial cells [38], and may be an important protective mechanism in ischemic injury. However, excessive IL-1 may be detrimental. IL-1β induces the expression of adhesion molecules, including intercellular adhesion molecule-1 and vascular cell adhesion molecule-1, on mesenchymal and endothelial cells [39-42]. IL-1 knockout (KO) mice and antagonist-treated rats develop significantly less infiltration of polymorphonuclear leukocytes, and have less severe renal histological and biochemical derangement, in ischemia-reperfusion (I-R) injury [43-45]. Deficiency or neutralization of IL-1 confers a similar protective effect in experimental glomerulonephritis (GN) [46-48].

Excessive tissue destruction may be mediated in part by IL-1α. Unlike IL-1β, IL-1α is active in its precursor form. This active precursor is constitutively expressed in epithelial cells [49] and the inflammatory resulting from cell necrosis may be mediated by surface IL-1α [29]. Activity of IL-1α is controlled by endogenous expression of intracellular IL-1Ra, which prevents signal transduction [50], consistent with findings in a model of renal I-R injury: the number of apoptotic tubular cells was lower in IL-1RA-treated animals 24 h after ischemia, which was paralleled by a Bax/Bcl-2 mRNA ratio towards anti-apoptotic effects [45]. Biologically active IL-1α is also expressed on the membrane of monocytes and B-lymphocytes [51,52]. In addition, the induction of many genes by IFN-gamma (INF-γ), including HLA-DR, ICAM-1, IL-18BP, and genes mediating its antiviral activity, depends on basal IL-1α but not IL-1β [53].

IL-18 (previously known as INF-γ inducing factor) is a member of the IL-1 cytokine family, with many properties distinguishing it from IL-1α and IL-1β. IL-18 is primarily expressed by macrophages and dendritic cells, but also by epithelial cells throughout the body [54,55]. One of the key features of IL-18 is its ability to induce INF-γ production [55] and subsequent T cell polarization [56,57]. IL-18 plays an important role in the TH1 response, primarily by its ability to induce IFN-γ production in T cells and natural killer cells [58]. Fas ligand-mediated cell death is also IL-18-dependent [59,60], and IL-18 neutralization is associated with a reduction in renal tubular apoptosis in unilateral ureteric obstruction (UUO) and I-R injury [60,61]. As well as to these distinguishing features, IL-18 also shares properties with other cytokines, including increases in cell adhesion molecules and chemokines, and NO synthesis [62-65]. IL-18 deficiency or neutralization is associated with decreased immune cell infiltration and relatively preserved renal function in UUO, I-R injury, and GN [61,66-68].

### Cell death and pyroptosis

Caspase-1 activation and subsequent production of IL-1β and IL-18 has a biphasic effect; low levels cause cytokine production but, above a certain threshold, can lead to pyroptosis [69]. This is a catastrophic form of cell death commonly found in monocytes, macrophages and dendritic cells, with morphological characteristics of apoptosis and necrosis. Cell lysis occurs due to caspase-1-dependent pore formation in the cell membrane, disruption of the cellular ionic gradient, osmotic driven water influx, and cell swelling [6,7]. This leads to inflammasome activation, release of proinflammatory cytokines, damaged DNA, and metabolic enzymes and, ultimately, cellular disruption releasing other DAMPS. Release of mitochondria into the extracellular space results in discharge of ATP that acts as a DAMP.

An alternative mechanism of cell death relates to activation of the P2X_7_R. Here, irreversible pore formation allows the non-selective passage of ions and hydrophilic solutes of up to 900 Da, resulting in colloido-osmotic cell lysis [33]. P2X_7_R-induced shrinkage depends on K^+^ efflux via K_Ca3.1_, a voltage-independent potassium channel activated by intracellular calcium, and a pathway of Cl^-^ efflux distinct from that implicated previously in apoptosis [70].

### Regulation of the inflammasome

Activation of the inflammasome results in a rapid and substantial inflammatory response. As such, the inflammasome is tightly regulated at both transcriptional and post-transcriptional levels. Basal expression of inflammasome components, in particular NLRP3, is relatively low [8]; pro-apoptotic pathways, such as FAS ligand-receptor interactions, are required to induce expression of ASC [9]. The subcellular location of inflammasome components facilitates its regulation. ASC is localized to the nucleus in quiescent cells, but it is recruited to the cytoplasm on cell activation [10].

Alternatively spliced inflammasome components generate protein variants with different activities. ASC has at least three different isoforms, one of which has an inhibitory effect on inflammasome activity [71]. Several proteins regulate inflammasome activity by sequestration of inflammasome components. Anti-apoptotic Bcl-2 proteins, including Bcl-2 and Bcl-x_L_, interact with NLRP1 to prevent ATP binding and inflammasome activation [12]. The pyrin-only proteins (POP) and the pyrin-containing NOD (PYNOD) proteins inhibit inflammasome formation via inhibition of NFκB and suppression of ASC, respectively [72,73]. Other inhibitory proteins include COP (CARD only protein), INCA (inhibitory CARD), and ICEBERG all three proteins contain a CARD and they are believed to act as decoys inhibiting formation of an active inflammasome [74].

### Drugs modulating the NLRP3 inflammasome /IL-1/IL-18 axis

Growing evidence suggests that the inflammasome and the IL-1β/IL-18 axis play an integral part in the pathogenesis of many acute and chronic conditions, including gout, rheumatoid arthritis, atherosclerosis, Alzheimer’s disease, diabetes mellitus, and, most recently, oxalate crystal nephropathy. Several components of the NLRP3 inflammasome have been implicated in renal disease (Table 2). Therapeutic interventions that modulate this pathway are being developed, and the functional significance of the inflammasome and the IL-1β/IL-18 axis in renal disease is of growing interest. Drugs inhibiting IL-1, P2X_7_R, and caspase-1 have been developed, although to date only IL-1 inhibitors have been successful in clinical studies of rheumatoid arthritis (RA) and cryopyrin-associated periodic syndrome (CAPS).

**Table 2:** Inflammasome and inflammatory renal diseases

**P2X7**	**Disease**	**Species**	**Antagonist/genetic deletion**	**Effect**	**Renal localization of inflammasome component**
Harada [75]	TNF-α stimulation	Rat	-	NA	Mesangial cells
Gonclaves [76]	Unilateral ureteric obstruction	Mouse	P2X_7_^−/−^	Beneficial	PTEC
Vonend [77]	Hypertension	Rat	-	NA	Glomerular podocytes
	Diabetes mellitus				
Turner [78]	Experimental glomerulonephritis	Mouse	-	NA	Glomeruli and infiltrating macrophages
		Rat			
					Glomeruli
	Lupus nephritis	Humans	-	NA	Glomeruli
					PTEC
Taylor [70]	Experimental glomerulonephritis	Rat	Antagonist	Beneficial	-
		Mouse	P2X_7_^−/−^		
**NLRP3**	**Disease**	**Species**	**Antagonist/genetic deletion**	**Effect**	**Renal localization of inflammasome component**
Deplano [79]	Glomerulonephritis	Rat	Genetic susceptible strain (cf. Protected strain)	NA	Glomeruli and bone marrow derived macrophages
Vilaysane A [80]	Non-diabetic acute and chronic kidney diseases	Human	NA	NA	PTEC
Vilaysane A [80]	Unilateral ureteric obstruction	Mice	NLRP3^*−/−*^	Beneficial	PTEC
Iyer S [81]	Ischaemia- reperfusion injury	Mice	NLRP3^*−/−*^	Beneficial	-
Jalilian [82]	None	Dog	NA	NA	Epithelial cells
**IL-1**	**Disease**	**Species**	**Antagonist/genetic deletion**	**Effect**	**Renal localization of inflammasome component**
Yamagishi H [83]	Unilateral ureteric obstruction	Mouse	IL-1 RA	Beneficial	PTEC
Haq M [44]	Ischaemia- reperfusion injury	Mouse	IL-1 RA	Beneficial	-
			IL-1R ^−/−^		
Chen A [46]	IgA nephropathy	Mice	IL-1 RA	Beneficial	-
Matsumoto [84]	Glomerulonephritis	Human	NA	NA	
Tam [85]	Glomerulonephritis	Rat	NA	N/A	
Lan [86]	Glomerulonephritis	Rat	IL-1RA	Beneficial	
Karkar [87]	Glomerulonephritis	Rat	Antibody	Beneficial	
Karkar [88]	Glomerulonephritis	Rat	IL-1RA and soluble IL-1R	Beneficial	
Tam [89]					
Timoshanko JR [48]	Crescentic glomerulonephritis	Mice	IL-1β ^−/−^	Beneficial	-
			IL-1R ^−/−^		
Lichtnekert [47]	Anti- GBM disease	Mice	NLRP3 ^−/−^	No effect	Renal dendritic cells
			Caspase1 ^−/−^	No effect	
			ASC ^−/−^	No effect	
			IL-1R1 ^−/−^	Benefit	
			Il-18 ^−/−^	Mild benefit	
Schorlemmer H [90]	SLE-like disease	Mice	IL-1 RA	Beneficial	-
Furuichi [43]	Ischaemia- reperfusion injury	Mice	IL-1αβ ^−/−^	Beneficial	glomeruli and cortical arterioles
			IL-1RA ^−/−^		
Rusai [45]	Ischaemia- reperfusion injury	Rats	IL-1 RA	Beneficial	-
Granfeldt [91]	Endotoxaemia	Pigs	NA	NA	Endothelial cells of the cortical arterioles were positive for IL-1β
					IL-1ra was detected in the glomerulus and tubular cells
Hertting [92]	E.Coli pyelonephritis	Mice	IL-1β ^−/−^	Harmful	-
**Caspase-1**	**Disease**	**Species**	**Antagonist/genetic deletion**	**Effect**	**Renal localization of inflammasome component**
Homsi [93]	Glycerol- induced AKI	Rats	Caspase-1 inhibitor	Beneficial	Constitutive tubular expression of IL-18
					Induction of tubular IL-1β
Wang [94]	Endotoxaemia	Mice	Caspase 1 ^−/−^	Beneficial	-
			IL-1 Ra	No effect	
			IL-18 antiserum	No effect	
Gauer [95]	None	Humans	NA	NA	Collecting duct alpha- and beta-intercalated cells express P2X_7_, IL-18
Edelstein [96]	Hypoxia	Mice	Caspase 1 ^−/−^	Beneficial	IL-18 in PTEC
			IL-18 binding protein	No effect	
**IL-18**	**Disease**	**Species**	**Antagonist/genetic deletion**	**Effect**	**Renal localization of inflammasome component**
Bani-Hani A [68]	Unilateral ureteric obstruction	Mice	Transgenic mice overexpressing human IL-18-binding protein	Beneficial	TECs
Wu H [66]	Ischaemia- reperfusion injury	Mice	IL-18 ^−/−^	Beneficial	TECs
			IL-18 ^−/−^ BM chimera	Beneficial	
			IL-18-binding protein	Beneficial	
Sugiyama M [67]	Bovine serum albumin-induced glomerulonephritis	Mice	IL-18R ^−/−^	Beneficial	-
Kinoshita K [97]	Autoimmune disease	Mice	IL-18R ^−/−^	Beneficial	-
Wang [61]	Ischaemia-reperfusion injury	Rat	IL-18-binding protein	Beneficial	-
Zhang [60]	Unilateral ureteric obstruction	Mice	Overexpress human IL-18-binding protein isoform *a*	Beneficial	-
VanderBrink [98]	Unilateral ureteric obstruction	Mice	IL-18 ^−/−^	NA	TECs
**ASC**	**Disease**	**Species**	**Antagonist/genetic deletion**	**Effect**	**Renal localization of inflammasome component**
Iyer S [81]	Ischaemia- reperfusion injury	Mice	ASC ^−/−^	Beneficial	-

*Abbreviations*: *PTEC* Proximal tubular epithelial cells, *NLRP3* Nod-like receptor protein 3, *IL-1RA* Interleukin 1 receptor antitagonist, *ARF* Acute renal failure, *TEC* Tubular epithelial cell, *BM* Bone marrow.

#### IL-1 inhibitors

The clinical application of IL-1 inhibitors has been slow, because the first generation of inhibitors, the recombinant IL-1 receptor antagonists, has a short circulatory half-life and limited affinity for the IL-1 receptor. A large molar excess of recombinant IL-1ra is needed to antagonize endogenous IL-1 effectively.

Drugs inhibiting the action of IL-1 include recombinant human IL-1ra (Anakinra), a humanized monoclonal IL-1β antibody (Canakinumab), and a neutralising antibody against IL-1α and IL-1β (Rilonacept). Anakinra competitively inhibits binding of IL-1 to the IL-1 receptor and has been successfully used in RA [99] and autoinflammatory syndromes [100]. Rilonacept is a dimeric protein consisting of the extracellular portion of the IL-1 receptor and the Fc portion of human IgG_1_[101]; it works by effectively neutralizing IL-1α and IL-1β. Preliminary data suggest it may be beneficial in patients with autoinflammatory syndromes [102,103]. Canakinumab, a monoclonal antibody against IL-1β, has a longer half-life compared with the other antagonists, and may be useful in patients with RA and CAPS [104,105]. Other diseases that may benefit from IL-1 blockade include acute gout [106], diabetes mellitus [100], inflammatory lung disease [107], adult-onset Still’s disease [108], and juvenile idiopathic arthritis [109].

#### P2X_7_R antagonists

Drugs inhibiting the P2X_7_R are currently in Phase 1 and 2 clinical trials [110]. At present there are no data to demonstrate a beneficial effect of P2X_7_R antagonism, although trials are still at an early stage. Preclinical data suggest P2X_7_R antagonists have a potential role in the treatment of inflammatory rheumatological [111,112], renal [78,113], and pulmonary diseases [114-116]. Although Phase 1 and 2 studies have demonstrated safety, preliminary studies have so far not shown clinical efficacy in the management of RA [117].

#### Caspase-1 inhibitors

Small molecule inhibitors of caspase-1 have been used in experimental models. Only pralnacasan (VX-740) and VX-765 have been used so far in patients; however, concerns about liver toxicity with prolonged use of pralnacasan have resulted in discontinuation of clinical trials in RA, psoriasis, and osteoarthritis [118]. A Phase 2 clinical trial of VX-765 (NCT00205465) has been completed, although the results have yet to be published [118].

### The inflammasome in renal disease

There is a better understanding of the role of IL-1 and IL-18 in renal disease, although the importance of the inflammasome in the activation and secretion of IL-1β and IL-18 has only been investigated recently. Several primary renal diseases are associated with NLRP3 inflammasome activation. Similarly, many systemic diseases affecting the kidneys are associated with NLRP3 inflammasome/IL-1β/IL-18 axis activation (Table 2). These include UUO [68,76,80,83], I-R injury [43-45,61,66,81], GN [46-48,67,70,90,97,119], sepsis [91,92,94], CKD [80,120], hypoxia [96], glycerol-induced renal failure [93], and crystal nephropathy [121]. Apart from two studies of CKD of various aetiologies [77,80] most of the disorders studied have been acute inflammatory diseases. Recent data suggests that the NLRP3 inflammasome is the principle cause of progressive renal failure in oxalate nephropathy [122]. P2X_7_R, IL-1β, IL-18, caspase-1, ASC, and NLRP3 are all associated with renal inflammation and injury (Table 2). Virtually every experimental model using genetic deletions and/or receptor antagonists/antiserum against the NLRP3 inflammasome pathway has shown decreased severity of disease, although publication bias cannot be excluded.

However, the functional significance of the inflammasome remains unclear in certain conditions. For instance, conflicting data exists with respect to ischaemia reperfusion injury. Whilst some reports describe a protective effect of IL-1 receptor blockade with Anakinra in ischemia-reperfusion injury [45,81], others demonstrate no benefit on renal injury responses [123]. This may be due to NLRP3 mediated injury that is independent of inflammasome activity [123]. In such circumstances, pharmacological inhibition of downstream targets may be less effective.

Intrinsic renal cells express components of the inflammasome pathway (Table 2). This is most prominent in tubular epithelial cells and, to a lesser degree, in glomeruli. The precise mechanisms involving the NLRP3 inflammasome in disease relate to both systemic and local (renal) activation. Limited studies using global knockouts and bone marrow chimeras suggest that systemic production of cytokines may have a greater effect on renal injury [66]. Findings related to genetic deletion or inhibition of the NLRP3 inflammasome pathway includes decreases in local cytokines and chemokines, inflammatory cell infiltrate, and apoptosis. It remains likely that locally released DAMPs result in inflammasome activation, resulting in chemokine release and immune cell infiltration. Differences in immune cell regulation of the inflammasome affect the susceptibility and severity of autoimmune GN [79].

The role of NLRP3 inflammasome activation in human renal disease is still uncertain. Consistent with experimental data P2X_7_R and NLRP3 are upregulated in lupus nephritis and non-diabetic CKD, respectively [80,119]. The most extensively studied component of the NLRP3 inflammasome in relation to renal disease is IL-18. Collecting duct alpha- and beta-intercalated cells express P2X_7_R and IL-18 under basal conditions [95]. An elevated serum IL-18 correlates with the development of diabetic nephropathy [124], while urine IL-18 is elevated in acute kidney injury associated with critical illness [125], cardiac surgery [126], and radiocontrast [127], supporting the notion that the inflammasome is intimately involved in wider inflammatory renal disease. Further studies investigating the NLRP3 inflammasome pathway in human disease are needed.

### Chronic kidney disease and inflammation

In addition to mediating acute forms of renal injury and disease, the IL-1/IL-18 axis may also be responsible for development of CKD itself and its related complications. Accelerated atherosclerosis and vascular calcification is a hallmark feature in CKD [128]. Vascular inflammation plays a role in vascular calcification and IL-18 may have a distinct role in mediating vascular injury among patients with advanced kidney disease. Basal levels of IL-18 are elevated in patients on maintenance haemodialysis [129]. The mechanism behind increased IL-18 production may relate to elevated levels of circulating MCP-1 in patients with CKD [130]. IL-18, through production of INF-γ, results in inflammation-related vascular injury, atherosclerotic plaque formation, and plaque instability [131-133]. IL-18 levels correlate with aortic pulse wave velocity [134], a surrogate for aortic stiffness and a predictor of major adverse cardiovascular events among patients with CKD [135].

In addition to cardiovascular disease, sepsis accounts for the majority of critical care admissions and mortality among patients with end-stage kidney disease [136]. The underlying mechanism(s) behind the increased susceptibility to sepsis relates in part to altered levels of IL-1 and IL-1RA, and monocyte activity. Basal levels of IL-1β, TNFα, and IL-6 are elevated in CKD and in dialysis patients [137]. The IL-1ra/IL-1β ratio is also elevated [137,138]. A higher IL-1ra/IL-1β ratio may participate in the complex immune disturbances by reducing the biological activity of this vital pro-inflammatory cytokine in playing a major role in the immune and inflammatory network.

Complications associated with CKD are clearly multifactorial and a greater understanding of the role of the NLRP3 inflammasome/IL-1/IL-18 axis in mediating these complications is required before any therapeutic strategy can be developed and applied.

## Conclusions

The NLRP3 inflammasome is becoming increasingly recognized as integral to the pathogenesis of many renal diseases and their complications. However, much of our knowledge of the inflammasome is limited to experimental models, but we need to elucidate its role in human renal disease, especially in CKD and its complications. Moreover, apart from inhibitors of IL-1, therapeutic agents targeting the NLRP3 inflammasome pathway suitable for use in humans are still lacking. Yet the inflammasome is likely to prove to be key pathogenic mechanism in nephrology and should be the subject of more intensive research.

## Abbreviations

AIM2: Absent in melanoma 2; ARF: Acute renal failure; BM: Bone marrow; CARD: Caspase recruitment domain; CPPD: Calcium pyrophosphate dehydrate; DAMP: Damage-associated molecular pattern; FIND: Domain with function to find; IFI16: Interferon-γ inducible protein 16; IL-1RA: Interleukin 1 receptor antagonist; LPS: Lipopolysaccharide; LRR: Leucine rich repeat; MDP: Muramyl dipeptide; MSU: Monosodium urate; NACHT: Nucleotide-binding and oligomerization domain; NAIP: NLR family apoptosis inhibitor; NLR: Nod-like receptor; NLRP3: Nod-like receptor protein 3; PAMP: Pathogen-associated molecular pattern; PRR: Pattern recognition receptor; PTEC: Proximal tubular epithelial cells; PYD: Pyrin domain; ROS: Reactive oxygen species; TEC: Tubular epithelial cell; TLR: Toll-like receptor.

## Competing interests

Clare M Turner

None declared

Nishkantha Arulkumaran

Wellcome Trust pre-doctoral training fellowship

Mervyn Singer

None declared

Robert J Unwin

Consultancy with AstraZeneca

Frederick Tam

Research project grants from Roche Palo Alto, AstraZeneca Limited, Cyclacel Limited and Baxter Biosciences

Consultancy for Roche Palo Alto and Baxter Biosciences

## Authors' contributions

CT, NA, Writing manuscript, Figures, Tables. Writing manuscript, Tables. MS, RJU, FT, Editing manuscript. All authors read and approved the final manuscript.

## Pre-publication history

The pre-publication history for this paper can be accessed here:

http://www.biomedcentral.com/1471-2369/15/21/prepub
